# Using commonly-available technologies to create online multimedia lessons through the application of the Cognitive Theory of Multimedia Learning

**DOI:** 10.1007/s11423-022-10181-1

**Published:** 2022-12-19

**Authors:** Thomas M. Cavanagh, Christa Kiersch

**Affiliations:** 1 Barowsky School of Business, Dominican University of California, 50 Acacia Ave, San Rafael, CA 94901 USA; 2 University of Wisconsin La Crosse, La Crosse, WI USA

**Keywords:** Cognitive Theory of Multimedia Learning, E-learning, Online multimedia lessons

## Abstract

Principles derived from the Cognitive Theory of Multimedia Learning (CTML; Mayer in: Multimedia learning, Cambridge University Press, Cambridge, 2021) provide valuable guidance for enlisting commonly-available technologies to create effective online multimedia lessons. Specifically, CTML can guide instructional designers on the use of slide-sharing programs to create concise, narrated animation segments; the use of survey programs to interpolate questions and prompts between these segments to facilitate generative learning activities; and the use of video-sharing sites to provide learners with control over relatively superficial aspects of instruction. The application of CTML to the design of online multimedia lessons raises a number of theoretical and practical questions, including the need to better understand the relationship between working memory capacity and working memory duration, the importance of retrieval as a learning process, and the relative impact of selection and organization processes on learning.

## Introduction

Designing online courses became a necessary skillset for educators across the world during the initial stages of the COVID-19 pandemic (United Nations Educational, Scientific, and Cultural Organization [UNESCO], 2021). Although in some ways this change can be seen as abrupt, it can also be interpreted as the continuation of a trend regarding the popularity of online learning. In the United States, for example, the number of primary and secondary school students enrolled in online programs was growing even before the pandemic began (Bettinger & Loeb, 2017). Online tertiary education, with varying degrees of reputability, has been available for decades and, more recently, many programs have begun offering “hybrid” structures, which combine both in-person and online learning (Allen & Seaman, 2014; Arbaugh, 2008; Kentnor, 2015; Means et al., 2013).

Online learning has broadened educational opportunities for non-traditional students who must balance education with work and family responsibilities, or who live in areas far from educational institutions (Arbaugh, 2005; Lemak et al., 2005). Online learning is more than a convenience, however. Research shows that well-designed online instruction leads to similar, if not better, learning outcomes as well-designed in-person instruction (Means et al., 2013; Sitzmann et al., 2006). It also comes with a host of benefits compared to traditional classroom environments, including features that allow students to access learning materials when and where it is most convenient for them (Klein et al., 2006; Sitzmann et al., 2006).

Like in-person instruction, online learning must be well-designed to be effective (Sitzmann et al., 2006), yet many educators never receive training specifically for creating online learning materials. This lack of training is exacerbated by the number of tools available for designing online course materials, overwhelming instructors with options. In addition to these technological options, research on online learning has been prolific, so much so that keeping up-to-date with the ever-growing number of learning principles can be overwhelming as well.

The Cognitive Theory of Multimedia Learning (CTML; Mayer, 2021) is well-suited to guide educators in the creation of online learning materials, as it is a theoretical framework designed with practical applications in mind. This conclusion is evidenced by the commonly-referenced ‘Mayer's Principles,’ or practical guidelines for putting CTML into practice in authentic learning/instructional settings. Yet, the intention should not only be to have CTML guide practice, but to foster a reciprocal relationship between the theory and practice. Such a relationship requires instructional practices to grow and respond to advances in the science (i.e., to put Mayer’s Principles into practice) and also requires the theory to grow and respond to how learning and instruction occur in authentic situations (e.g., Kuba et al., 2021).

The purpose of this article is to reinforce a reciprocal relationship between CTML and practice by reviewing and summarizing practical recommendations from this theoretical framework as applicable to online multimedia lessons in authentic adult learning environments, while also highlighting limitations of CTML that arise when attempting to apply this theory. Our contribution to practice is enhanced by our inclusion of *how* instructors may incorporate CTML principles using commonly available software programs and websites, informed by our own experience teaching online undergraduate and graduate-level courses. We contribute to the empirical work in multimedia learning through discussion of the theoretical gaps that arise through application of CTML, including the need to better understand the relationship between working memory capacity and working memory duration, the importance of retrieval as a learning process, and the relative impact of selection and organization processes on learning. We conclude with the identification of opportunities to further strengthen the reciprocal relationship between CTML and practice, including future research directions and a need to more fully consider the role of student satisfaction in the learning process. Specifically, the following inquiries will guide our critical review:

- What CTML principles are clearly supported by research and practically useful?
- How can CTML principles be applied using commonly available online instruction/learning tools?
- What limitations exist when using CTML to guide the development of online multimedia lessons?
- What gaps exist in the current research surrounding CTML that would be most beneficial for practitioners?

We begin with an overview of CTML (e.g., Mayer, 2021) in which we highlight the key assumptions from the theory as relevant to the creation and delivery of effective online instruction. Taking inspiration from Mayer (2021) and Sung and Mayer (2012), we refer to instructional materials as “online multimedia lessons,” where “lessons” refers to a presentation designed to foster learning, “online” refers to the lesson being delivered over the internet, and “multimedia” refers to the use of both words and pictures.

## Cognitive Theory of Multimedia Learning

**Fig. 1:**
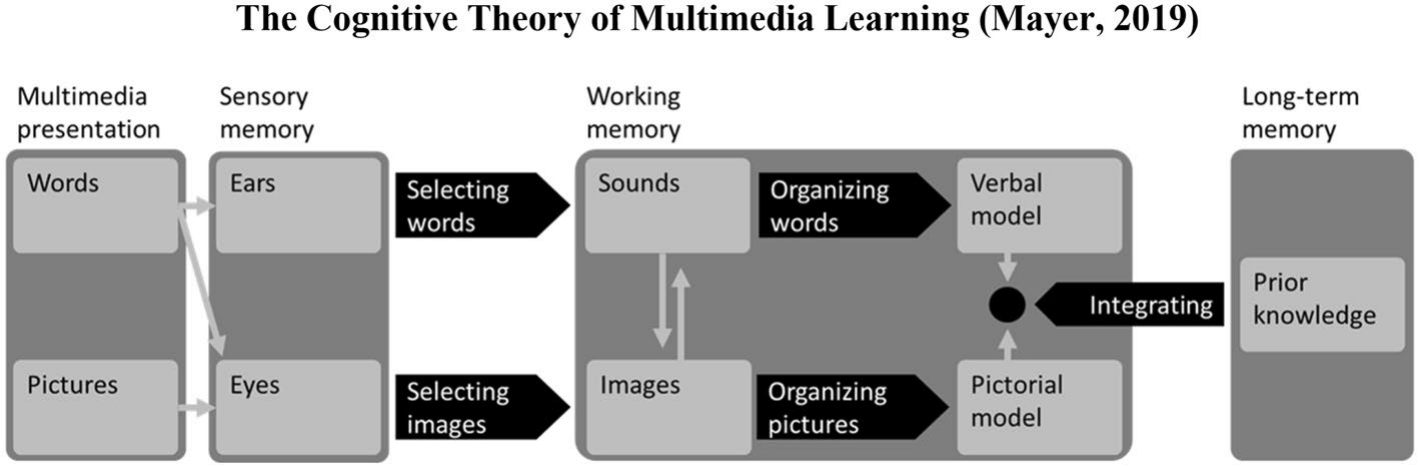
The Cognitive Theory of Multimedia Learning (Mayer, 2019)

The Cognitive Theory of Multimedia Learning (CTML; see Fig. 1) offers a robust theoretical framework to guide the design of online multimedia lessons (Mayer, 2017, 2021).

CTML is based on three main assumptions: the active processing assumption (learners are active participants in acquiring new knowledge); the limited capacity assumption (learners have limited cognitive capacity for processing information in working memory); and the dual-channel assumption (learners have two independent channels for processing information) (Mayer, 2021). We explain each of the three assumptions below.

### Active processing assumption

The active processing assumption states that learners actively engage with the learning environment, selecting relevant information, organizing it in working memory, and integrating it with existing information from long-term memory (Mayer, 2021). This assumption is so important to CTML that effective learning environments can essentially be defined as environments that facilitate the three processes of selection, organization, and integration (Abeysekera & Dawson, 2015; Klein et al., 2006; Mayer, 2021).

### Limited cognitive capacity assumption

Learners must actively engage in learning processes in order to acquire new knowledge, but their ability to do so is limited by their cognitive capacity. This premise is referred to as the limited cognitive capacity assumption, which states that learners have limited cognitive capacity for representing and organizing information in working-memory (Mayer, 2021; Sweller, 2020). Research in cognitive psychology estimates this capacity at approximately four, plus or minus one, chunks of information, where “chunk” refers to elements of information that are distinct enough from one another to be considered unique but similar enough to be grouped together in a meaningful way (Cowan, 2001; Miller, 1956; Oberauer et al., 2018).

### Dual channel assumption

Cognitive capacity is limited, but it can be maximized through application of the dual-channel assumption, which states that learners have two independent processing channels, each with their own capacity (Mayer, 2021). One of these channels processes verbal information, such as text and narration, while the other processes nonverbal information, such as pictures and animations (Paivio, 1969). Well-designed multimedia lessons take full advantage of both channels, for example, by combining narration and animation during instruction (Mayer, 2021).

In sum, CTML states that learners actively engage in selecting, organizing, and integrating information, that the amount of information learners can process at any given time is limited, and that cognitive architecture includes independent verbal and nonverbal channels for processing this information. Over a dozen learning principles have been developed and tested based on these three assumptions (Mayer, 2021). In the next section, we explore our first two inquiries by discussing how to apply a subset of these principles to create online multimedia lessons. Specifically, the broader research on CTML suggests that instructors should:

- Create concise, narrated animations that utilize both verbal and nonverbal information to explain concepts
- Divide concise, narrated animations into user-paced segments
- Separate narrated animation segments with questions and prompts to encourage learners to engage in generative learning activities
- Provide learners with control over superficial aspects of instruction.

We will describe each of these principles and how they can be applied using commonly available online instruction/learning tools.

## Practical applications of CTML principles

### Concise, narrated animations

In the traditional classroom, instruction is often built around live lecture accompanied by prepared slides, maps, or a whiteboard for writing notes and formulas. In online learning environments, instructors often record themselves as they talk over visual aids. Within the context of CTML, these types of videos are referred to as concise, narrated animations (CNAs), and evidence shows that, when they are well-designed, they can be an effective instructional tool (Mayer, 2021). Below we will discuss how to create effective CNAs by including words and pictures (multimedia principle), eliminating decorative elements (coherence principle), avoiding on-screen text that is redundant with narration (redundancy principle), syncing related elements visually and temporally (spatial and temporal contiguity principles), highlighting key information (signaling principle), and narrating in a conversational tone (personalization principle).

#### Multimedia principle

Perhaps the most important principle for creating effective CNAs is the multimedia principle, which states that people learn more effectively when instructional materials include both words and pictures, as opposed to words alone (Mayer, 2021). Due to the dual channel assumption, combining words and pictures facilitates the creation and integration of both verbal and nonverbal representations, leading to more meaningful learning than either mode alone (Mayer, 2021; Paivio, 1969).

**Fig. 2:**
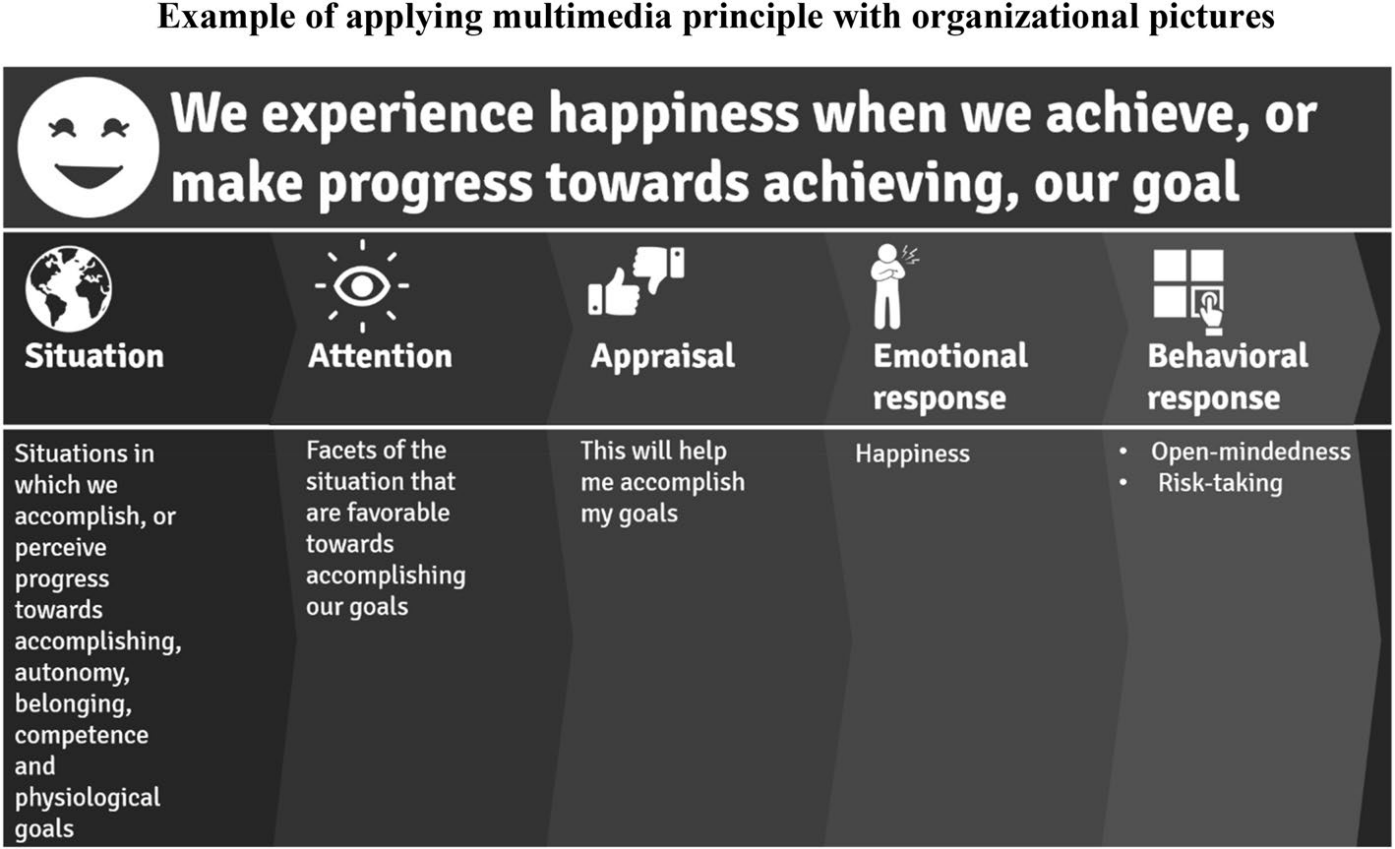
Example of applying multimedia principle with organizational pictures

When used for instruction, pictures can be broadly categorized into four types: decorative (pictures designed to catch learners’ attention without enhancing instructional content), representational (pictures that portray a single element discussed in narration), organizational (pictures that illustrate relationships among concepts), and explanative (pictures that illustrate how a system functions) (Levin & Mayer, 1993). The majority of pictures used in traditional textbooks are decorative or representational, which, unfortunately, are also the least effective types for enhancing learning (Mayer, 2021). Instructors should instead use organizational and explanative pictures explicitly to support and elaborate on the concepts communicated through narration. Common slide-sharing programs, such as PowerPoint, provide the necessary features to create these types of pictures, and to combine them with recorded narration. Figure 2 illustrates how organizational pictures can be created in a slide-sharing program, in this case to demonstrate a model of emotional experience.

#### Coherence principle

The coherence principle states that, regardless of whether it is delivered through words or pictures, information added to online multimedia lessons only facilitates learning if it is relevant to learning outcomes; irrelevant words and pictures undermine learning (Mayer, 2021). For example, in a lesson on lightning formation, an instructor might include a story about a person who was struck by lightning in an attempt to engage learners, even though the story is not directly related to learning goals. Instructors are often tempted to add interesting stories of this type, along with exciting pictures and decorative additions such as background music, to lessons in an attempt to grab and maintain learners’ attention. Unfortunately, these decorative additions (referred to as seductive details) have a significant negative impact on learning outcomes (Rey, 2012).

The negative impact of seductive details can be explained by the limited capacity assumption (Mayer, 2021). Learners are able to simultaneously process only a small amount of information, and relevant and irrelevant information compete for processing capacity. This situation is exacerbated when irrelevant information is specifically designed to be attention grabbing, as seductive details, by definition, are. Due to their effectiveness at competing for processing capacity, seductive details have a large, negative impact on learning outcomes (Rey, 2012).

#### Redundancy principle

The coherence principle provides guidance about what information to exclude from multimedia presentations; the redundancy principle extends that guidance, stating that on-screen text should not be redundant with narration (Mayer, 2021). Although on-screen text is visual and narration is auditory, both are processed in the verbal channel. At best, these unique streams of information are redundant, and the instructional designer has only wasted processing capacity that could have been invested in other learning materials. At worst, the information is not perfectly redundant (as, for example, when the learner reads ahead of the narrator), and the now-contradictory streams of information compete for limited verbal processing capacity. This competition for working memory capacity interferes with the selection, organization, and integration of information from instructional materials and undermines learning outcomes (Mayer, 2021).

The redundancy principle is not absolute, and there are times when redundant on-screen text is appropriate (Mayer & Johnson, 2008). Minimally redundant text should be used to highlight keywords, technical terms that learners may be unfamiliar with, terms that are not in the native language of the learner, and to provide abridged summaries of learning content (Adesope & Nesbitt, 2012; Mayer, 2021). When used in this way, text directs learners’ attention to the most important instructional information, facilitating effective selection (Mayer & Johnson, 2008).

#### Spatial and temporal contiguity principles

The coherence and redundancy principles focus mainly on accommodating limited processing capacity, but processing is limited in terms of duration, as well (Cowan, 2001; Mayer, 2021). If information is not consciously maintained in working memory, it tends to decay relatively quickly (Barrouillet et al., 2007). In online multimedia lessons, instructional designers can avoid this decay by placing related audio and visual components near one another in both space and time. This is referred to as the spatial contiguity and temporal contiguity principle, respectively (Mayer, 2021).

The spatial contiguity principle states that on-screen text should be placed in visual proximity to related animations (Mayer, 2021). For example, in an animation describing the function of a piston in a four-stroke engine, the instructor might decide to include on-screen text of the technical term “piston”; if so, it should appear in the animation right next to the visual depiction of the piston. A meta-analysis of 58 effect sizes covering 2426 observations revealed a large overall effect size for the impact of spatial contiguity on learning in multimedia environments, providing strong evidence supporting its use (Schroeder & Cenki, 2018). In regard to boundary conditions, spatial contiguity is less important when learners are already familiar with instructional content, when instructional content is simple, and when pictures and animation are easily understandable without text (Mayer, 2021).

Just as the spatial contiguity principle states that related words and pictures should be visually proximal, the temporal contiguity principle states that narrations and animations should be chronologically synced (Mayer, 2021). Syncing narration and animation ensures that verbal and nonverbal models can be processed simultaneously in working memory, before either has a chance to decay. Syncing verbal and nonverbal information in this way has a large effect on learning outcomes (Ginns, 2006).

**Fig. 3:**
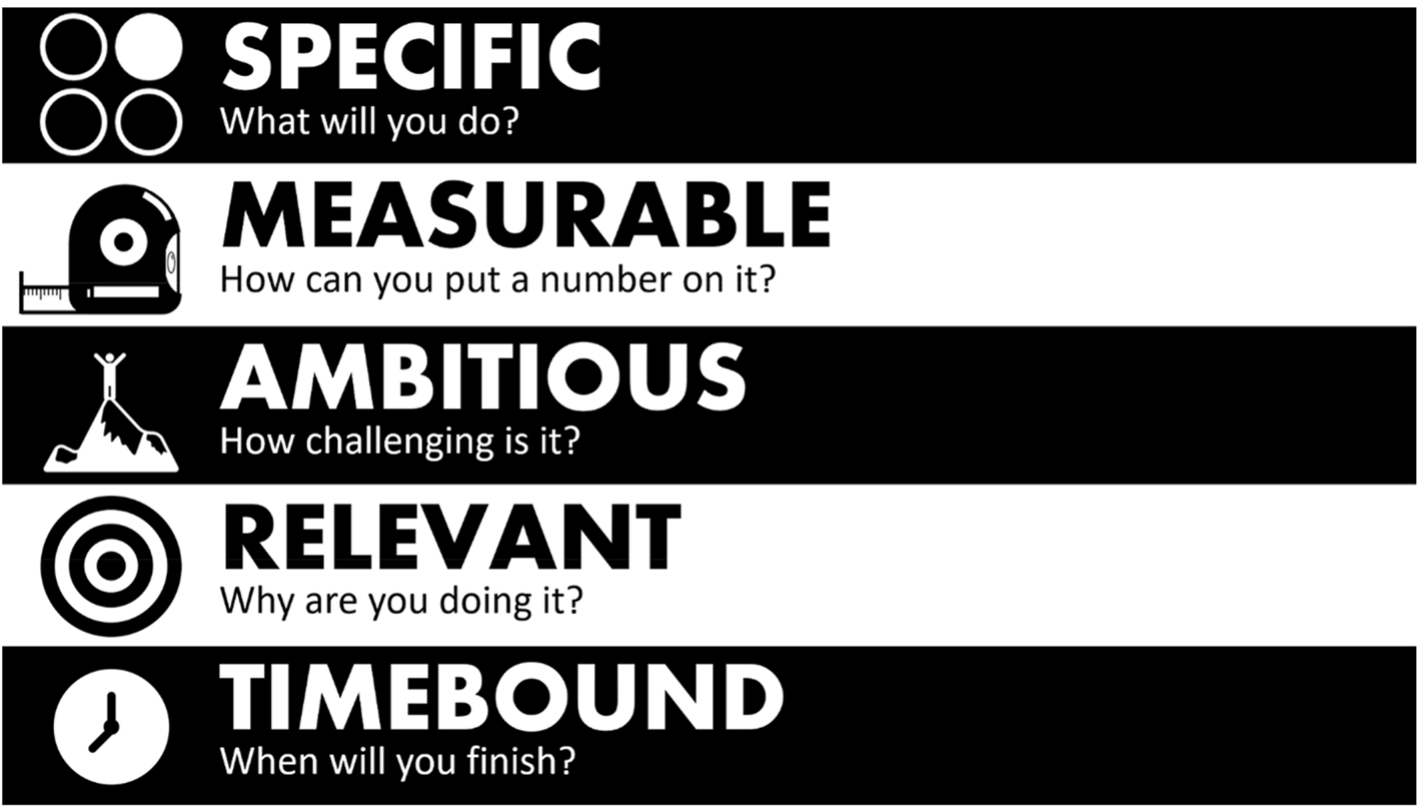
Example of applying spatial and temporal contiguity principles with animated graphics

Both the spatial and temporal contiguity principles are easy to apply within common slide-sharing programs like PowerPoint. In addition to features that allow the easy combination of text and pictures, these programs often have features to animate graphics in such a way so that relevant pictures appear in sync with narration. Figure 3 illustrates spatial and temporal contiguity principles in action. By specifying the animation within the slide-sharing program to group the graphics ‘one by one,’ the instructor can narrate each aspect of the SMART goal acronym as only the relevant icon image and text appear.

#### Signaling principle

Signaling (also referred to as “cueing”) refers to a number of techniques which call attention to relevant concepts contained in multimedia lessons, the organization of those concepts, and the relationships among them (De Koning et al., 2009; van Gog, 2014). Signals do not add new information to instruction, they simply draw attention to or repeat material in such a way as to facilitate selection and organization (Mayer, 2021).

Signals can be categorized into verbal and visual types (Mayer, 2021). Verbal signals facilitate the processing of verbal material, and can coordinate processing of corresponding words and pictures. Verbal signals include traditional methods such as outlining, the use of headings, and pointer words such as “first…second…third” that provide structure for text. Visual signals can include coloring of key elements in a graphic, pointing gestures, coordinating of verbal and visual cues (i.e., temporal contiguity), and the use of arrows, flashing graphics, and graying out of non-essential graphics.

A recent meta-analysis analyzing studies involving participants from elementary schools, high schools, vocational schools, and universities, found evidence for a small-to-medium effect of signaling on both comprehension and transfer (Richter et al., 2016). Signaling was more effective for low versus high prior knowledge learners and system versus self-paced instruction. The results provide strong evidence for the signaling effect, especially in online multimedia lessons.

#### Personalization principle

The principles discussed so far deal mainly with the content and structure of online multimedia lessons; the personalization principle deals with the tone of those lessons. Specifically, the personalization principle rejects the traditional formal style of expository text and narration in favor of a more casual, conversational style (Mayer, 2021). This personalized conversational style can be accomplished through the use of first- and second-person personal pronouns, direct statements to the learner, and polite requests instead of impersonal demands (Mayer, 2021). Personalization increases interest and outcomes by making learning content seem more relevant to learners, and activates sense-making processes associated with face-to-face social encounters (Ginns et al., 2013). Personalization has a medium to large effect on perceived friendliness of instruction (*k* = 5, *d* = .46, 95% CI [.22, .70]), a large effect on cognitive processing (*k* = 4, *d* = .62, 95% CI [.13, 1.11]), a moderate effect on retention (*k* = 30, *d* = .30, 95% CI [.18, .44]), and a large effect on transfer (*k* = 25, *d* = .54, 95% CI [.25, .83]) (Ginns et al., 2013).

In sum, instructors can transform traditional lectures into concise, narrated animations appropriate for online multimedia lessons by combining words and pictures, eliminating irrelevant information, reserving on-screen text for technical terms, coordinating elements in space and time, highlighting key information, and narrating in a casual style. These principles can be applied with features in commonly available slide-sharing programs, such as PowerPoint, and then exported as video files to be shared with learners on video-sharing sites, such as YouTube. In the next section we will discuss the segmenting principle, which states that CNAs should be divided into short, self-paced segments to ensure that learners have sufficient time and capacity to process instructional materials.

### Segmenting

The segmenting principle states that people learn better when online multimedia lessons are presented in short, user-paced segments rather than as a continuous unit (Mayer, 2021). Applied to CNAs, this means that instead of presenting narrated animations as a single unbroken video, it is more effective to divide them into segments that learners can progress through at their own pace (Mayer, 2021). A meta-analysis of 56 studies and 7713 participants found that, overall, segmenting positively impacted retention (*k* = 67, *d* = .032, 95% CI [.20, .43]) and transfer (*k* = 56, *d* = .36, 95% CI [.24, .48]), reduced cognitive load (k = 20, *d* = .23, 95% CI [.06, .39]), and increased time spent on learning (k = 19, *d* =  − .92, 95% CI [− 1.64, − .20]) (Rey et al., 2019). Interpreted through the lens of CTML, segmenting improves learning outcomes by accommodating limited cognitive capacity, providing a pause in instruction that allows learners to stop and process the information they have already received before moving on to more information (Spanjers et al., 2010).

The optimal length of segments has not been firmly established and remains an important research question (Mayer, 2021; Schacter & Szpunar, 2015). Ideal segment length likely depends on factors such as instructional content (for example, segments showing manual skills, such as a soccer play, might be very short, whereas segments focusing on abstract concepts might be longer), qualities of the learner (prior knowledge and working memory capacity), and characteristics of the delivery medium. Existing evidence combined with the limited capacity assumption suggests that segments should be as short as possible to cover necessary information.

### Interpolate questions between segments

Dividing narrated animations into concise, learner paced segments improves learning outcomes in and of itself; it also provides instructors with the opportunity to interpolate questions and prompts throughout instruction (Schacter & Szpunar, 2015). These questions and prompts can be used to encourage learners to actively engage in generative activities, that is, sense-making activities that involve selecting, organizing, and integrating information from instructional materials (Fiorella & Mayer, 2016; Mayer, 2021). There are a number of thorough reviews on the effectiveness of various generative activities (see especially Dunlosky et al., 2013; Fiorella & Mayer, 2016). Below we will review two types of generative activities which have demonstrated moderate-to-high utility, have been empirically tested in online learning environments, and which are fast and easy for most instructors to implement: practice testing and self-explanations.

#### Practice testing

Practice testing refers to the observation that retrieving information facilitates later retrieval of that information (Roediger & Karpicke, 2006). It specifically encompasses the use of low-stakes testing as a formative tool, as contrasted with high-stakes summative testing (Adesope et al., 2017; Dunlosky et al., 2013; Roediger & Karpicke, 2006). Examples of practice testing include using flashcards to memorize vocabulary, answering study questions embedded in a textbook, or solving practice problems at the end of a math book chapter (Dunlosky et al., 2013). Although some studies have examined the effects of practice testing on higher-level outcomes, the bulk of research has explored testing as a tool that facilitates recall (especially long-term recall) (Dunlosky et al., 2013; Karpicke & Zaromb, 2010). Within the framework of CTML, practice testing is hypothesized to help learners select the most relevant information from long-term memory, organize it by strengthening connections between related pieces of information, and integrate it with previous knowledge in long term memory (Fiorella & Mayer, 2016).

Empirically, there is overwhelming evidence for the positive impact of practice testing across conditions (e.g., lab vs. classroom), participants (e.g., preschool children to older adults), learning materials (e.g., word lists, paired associated, expository prose), practice testing materials (e.g., short-answer, free-recall, cued-recall, multiple-choice), and final testing materials, indicating that testing is an effective and practical pedagogical tool to implement in a variety of instructional settings (Dunlosky et al., 2013; Roediger & Karpicke, 2006). Two-recent meta-analysis have confirmed earlier results, finding specifically that practice testing is superior to both no-activity controls and restudy conditions (Adesope et al., 2017), that short answer and free recall practice tests are more effective than multiple-choice or recognition tests, that feedback increases the size of the effect, and that delayed feedback is more effective than immediate feedback (Rowland, 2014).

Practice testing has also been studied specifically within the context of multimedia learning, and evidence indicates that it remains effective in that environment (Johnson & Mayer, 2009). Most Learning Management Systems (LMS) include quiz and survey tools that can easily be applied to include practice testing for learners, as do freely available survey programs such as Google Forms. Learners’ responses can be automatically evaluated or learners can compare their answers to a key available at the end of the lesson, providing the feedback necessary to maximize the positive impact of practice testing and correct incorrectly encoded responses.

#### Self-explanations

Prompts can also be interpolated between CNA segments to encourage self-explanation, defined as a process through which learners generate inferences about instructional content, and then map those inferences onto existing mental models (Wylie & Chi, 2014). Self-explanation may involve elucidating how a system functions, the effect of each step in a procedure, the motives of a character in a story, or concepts from an expository text (Bisra et al., 2018). Whereas practice testing tends to focus on the recall of information presented during instruction, self-explanation requires learners to go beyond instructional materials, focusing on the why behind the information and, by doing so, connect it to already-learned-principles (Bisra et al., 2018; Chi et al., 1989; Fiorella & Mayer, 2016; Karpicke & Zaromb, 2010; Wylie & Chi, 2014). Although adept learners spontaneously self-explain, learners can be prompted to self-explain as well (Bisra et al., 2018; Fiorella & Mayer, 2016; Wylie & Chi, 2014).

From the perspective of CTML, self-explanation facilitates learning by supporting the processes of selecting, organizing, and integrating information presented during instruction (Fiorella & Mayer, 2016). When learners explain why a certain rule exists, or why certain steps follow in sequence, they must derive general principles from specific instances, facilitating the integration of new information with prior knowledge, and the transfer of learned information to novel situations (Bisra et al., 2018; Chi et al., 1989). Additionally, self-explanation helps learners identify gaps in understanding, guiding future learning efforts (Chi et al., 1989; Wylie & Chi, 2014). Empirical evidence provides strong support for the effectiveness of self-explanation on learning outcomes (Chi et al., 1989; Fiorella & Mayer, 2016; Wylie & Chi, 2014), including results from a recent meta-analysis by Bisra et al. (2018) that found a moderate overall effect size for self-explanation across grade levels, academic domains, instructional media, and learning outcomes.

To encourage self-explanation, instructors should use prompt types that align with the needs of the learner as well as the content, format, and goals of instruction. For example, prompts that encourage learners to make explicit connections between different sources of information (e.g., by prompting learners to provide a verbal explanation of a graphic model) are especially well-suited for multimedia instruction (Bisra et al., 2018; Wylie & Chi, 2014). The effectiveness of prompts is directly related to the quality of self-explanation that learners generate, so prompts should provide the amount of structure necessary to facilitate adequate self-explanations (Chi et al., 1989; Fiorella & Mayer, 2016; Wylie & Chi, 2014). In practice, this means that instructors should provide more structured prompts when learners’ previous knowledge is low, or when instructional information is complex (Wylie & Chi, 2014). Too much structure, however, can negatively impact learning outcomes, likely because it interferes with learners’ idiosyncratic understanding of instructional content (Bisra et al., 2018). Interestingly, providing instructor-generated explanations after prompting learners to self-explain undermines the effectiveness of self-explanation prompts, potentially because, knowing they will be provided with correct answers, learners put less effort into generating their own explanations (Bisra et al., 2018; Dunlosky et al., 2013).

In addition to prompt type, when prompts occur during instruction also impacts their effectiveness. Perhaps counterintuitively, Bisra et al. (2018) found that self-explanation prompts prior to instruction are more effective than those embedded throughout or placed at the end (although, regardless of when they were presented, prompts had a significant, positive impact on learning outcomes). One potential explanation for this finding is that self-explanation prompts prior to instruction encourage learners to activate existing cognitive models, and, once activated, those models can be adjusted and refined based on incoming information. An effective strategy is to prompt learners to explain a topic prior to instruction, prompt them to periodically update their explanation during instruction, and then revise their explanation at the end of instruction, highlighting how their understanding has changed (Bisra et al., 2018). This approach could be easily adapted to online multimedia lessons, such that learners are prompted to offer an explanation at the beginning of the lesson, asked to update relevant sections of that explanation at the end of each narrated, animated segment, and then prompted to revise their initial explanation at the end of the lesson. Table 1 provides an example of this approach, as applied in an online multimedia lesson on how to effectively deliver feedback, part of an online course on leadership.

**Table 1:** Example of using self-explanations before, during, and after a concise narrated animation

Time point	Example prompt
Before	Based on prior lessons from this class and assigned readings on this topic, what differentiates effective feedback from ineffective feedback in the workplace?
During	How do the feedback principles presented in this lesson compliment and/or contradict effective leadership principles you have already learned?
After	Considering what you have learned up until this point in the course, how should leaders apply effective feedback principles in various work contexts?

In sum, self-explanation prompts are an effective tool for facilitating learning, and, because they facilitate the organization of information from multiple sources, are particularly appropriate for application in multimedia instruction. Like practice test questions, LMS quiz or survey functions and freely available survey programs such as Google Forms allow instructors to easily incorporate self-explanation prompts between concise, narrated animation segments.

Now that we have reviewed how to create narrated, animated segments, and the value of interpolating questions and prompts between those segments, we will discuss the CTML principle of providing learners with control over how they engage with those learning materials.

### Learner control

Learner control refers to techniques that provide learners with the freedom to make decisions about how they engage with instructional materials (Kraiger & Jerden, 2007). This freedom can extend to relatively superficial aspects of the instruction, such as the pace of a multimedia video, and to deeper pedagogical aspects, such as the difficulty of material or the amount of feedback learners receive (Brown et al., 2016). Learner control, then, is not a single, unitary dimension, but a collection of tactics. These various forms of learner control interact with other aspects of the instructional environment, such as characteristics of learners themselves and the goals of instruction, making it difficult to make statements about the positive or negative effects of learner control on learning outcomes in general (Brown et al., 2016; Kraiger & Jerden, 2007).

Despite the complexity of learner control, and its interaction with other aspects of the instructional environment, several meta-analyses have provided relatively consistent results about which types of learner control work under which conditions. There is evidence that learner control over relatively superficial aspects of instructional delivery, such as pace, improves learning outcomes, whereas learner control over pedagogical aspects of the training, such as the difficulty of material or the amount of feedback, undermines learning outcomes (Brown et al., 2016; Kraiger & Jerden, 2007). Results also indicate that control facilitates performance for learners with high previous knowledge about a topic, but has no effect, or even negative effects, for learners with low previous knowledge (Carolan et al., 2014). Lastly, learner control improves performance on skill-based and transfer outcomes, but has a negligible or negative effect on recall and knowledge-based outcomes (Brown et al., 2016; Carolan et al., 2014; Kraiger & Jerden, 2007; Landers & Reddock, 2017).

Below we will discuss the benefits of providing learners with control over the pace of instruction and over the presence of captions.

#### Pace control

Pace control refers to learners’ ability to adjust the speed of instruction, for example, by skipping material they find easy and spending more time with material they find difficult (Kraiger & Jerden, 2007). Pace control is related to reduced mental effort and improved learning outcomes, especially in regard to complex learning materials (Hasler et al., 2007; Stiller et al., 2009, 2011). Pace control is especially useful for difficult concepts, demonstrating manual skills, and learners with slower processing speeds, such as older adults or neurodivergent individuals (Abeysekera & Dawson, 2015; Lents & Cifuentes, 2009; Wolfson et al., 2014).

Pace control has largely been studied in regard to the ability to pause and rewind learning materials, but available technology can also provide learners with the ability to increase the speed of narration. Research shows that increasing the speed of narration reduces total time spent on instruction, has no negative effect on learning, and, at least in some cases, improves learner satisfaction (Ritzhaupt et al., 2008). These findings make sense, given that the rate of speech in conversation generally hovers around 150 words per minute, yet people are able to process up to 300 words per minute with negligible loss of comprehension (Goldhaber, 1970). Thus, we recommend that, in addition to the ability to pause or rewind, instructors give learners the option of speeding up the pace of instruction.

#### Captioning

In addition to control over pace, learners should be given control over the appearance of captions in narrated learning materials. As mentioned above, the redundancy principle states that instructional designers should eliminate on-screen text that is redundant with narration, and captioning would seem to contradict this principle. Redundant on-screen text, however, can benefit learners with hearing impairments, learners who are non-native speakers of the language used in instruction, and learners who are unfamiliar with content-specific jargon (Abeysekera & Dawson, 2015; Wolfson et al., 2014). Captioning provides the option of redundant text to those learners who need it. Instructors should also keep captions in mind when designing multimedia materials; they should ensure that, if learners do decide to use captions, those captions do not obscure important learning information (e.g., by covering up on-screen animations or labels).

In sum, providing learners control over superficial aspects of instruction, such as pace, improves learning outcomes. Common video sharing websites, such as YouTube, provide instructional designers most of the tools they need to facilitate learner control. In addition to allowing learners to pause, rewind, and speed up the pace of videos, YouTube automatically captions videos, making it easy to give learners access to these features. An important caveat is that instructors should not take it for granted that learners are familiar with control features; if they want learners to use them effectively, they need to explicitly teach them how (Bell & Kozlowski, 2002).

### Summary of CTML applications

In addressing our first two inquiries, *What CTML principles are clearly supported by research and practically useful?* and *How can CTML principles be applied using commonly available online instruction/learning tools?*, we reviewed a number of empirically tested learning principles derived from CTML that can be applied to online multimedia lessons. These principles dictate instructors to create concise, narrated animations using slide-sharing programs; to divide those animations into shorter segments, export them as video files, and upload them to video-sharing sites; to embed those videos in survey programs, punctuated by questions and prompts to facilitate generative activities; and to take advantage of learner control features provided by video-sharing sites.

The application of CTML principles to online multimedia lessons reveals several limitations of this framework as well as gaps that currently exist in the research that, if filled, would be most beneficial for practitioners. This next section of the paper will explore such limitations and gaps, addressing inquiry 3 (*What limitations exist when using CTML to guide the development of online multimedia lessons?*) and inquiry 4 (*What gaps exist in the current research surrounding CTML that would be most beneficial for practitioners?*), ultimately providing guidance for future work of both scientists and practitioners of online learning/instruction.

## CTML practical limitations and research gaps

Now that we have reviewed learning principles derived from CTML and summarized how they may be applied in authentic online instruction settings, we will discuss a number of unaddressed theoretical questions that arise from applying these principles to online multimedia lessons. In doing so, the practice of online instruction may be applied to the strengthening of online learning science. Specifically, we suggest that CTML needs to clarify the relationship between working memory capacity and working memory duration, the importance of retrieval as a learning process, and the relative impact of learning principles on selection and organization processes. The application of CTML to authentic online instruction/learning scenarios further highlights the need for future research to consider outcomes beyond the traditional ‘learning and transfer,’ including learner satisfaction and engagement. We explore each of these current limitations of CTML as an unanswered question that may guide future research in this area.

### What is the relationship between working memory capacity and duration?

MAYER (2021) describes working memory as limited in both capacity and duration, and he devotes a substantial amount of time to discussing the nature and importance of the capacity limitation (“limited capacity” is one of the three main assumptions of CTML). The duration of working memory, however, is described in a single word: “short” (p. 42). Given that duration potentially plays a key role in working memory (Barrouillet et al., 2007; Grondin, 2001), the relationship between capacity and duration should be more fully understood. For example, one of the open questions regarding the segmenting principle is the ideal length of segments (Mayer, 2021). If we focus only on capacity limitations, then the length of a segment will be determined by the total amount of information included in that segment. If we consider duration limitations as well, then segments will be limited in regard to how much information is presented over what amount of time.

The relationship between capacity and duration limitations is likely complex. If learners are presented with too much information too quickly, it will overwhelm their capacity limitation and information will be lost; if learners are presented with the same amount of information over a much longer time period, however, the initial information might decay before all of the information is presented. Instructional designers need clear guidance to create segments that accommodate working memory limitations in both capacity and duration.

In addition to segment length, understanding working memory duration will also provide guidance in regard to increased narration speed. Given the strong focus on capacity limitations within CTML, we would expect increased narration speed to negatively impact learning outcomes, as it increases the amount of information presented to learners and overloads their limited cognitive capacity. Research, however, demonstrates the opposite: increasing narration speed by up to 1.8 times has no negative impact on learning (Ritzhaupt et al., 2008). This is not a minor point—nearly doubling the speed of instruction has the potential to vastly increase the efficiency of learning (learners are exposed to twice as much information in the same amount of time), and potentially represents an enormous advantage for recorded multimedia instruction compared to traditional instruction (when teaching in person, instructors are limited in their ability to increase their narration speed beyond a comfortable rate of speaking). Increasing narration speed might not just help learners progress through instructional materials more quickly, however; it might help them learn the material better by reducing the amount of information that decays over the course of a given segment.

These are compelling propositions, and theoretically elaborating the nature of the relationship between working memory capacity and duration could provide valuable guidance for instructional designers. Research on working memory duration already exists in cognitive psychology (see especially Oberaur et al., 2018), and explicitly incorporating this research into CTML provides an opportunity for theoretical development.

### Does retrieval need to be incorporated into CTML as a key learning process?

In addition to the nature of the duration of information in working memory, CTML lacks an explicit description of the cognitive process of retrieval. This oversight is especially relevant in regard to explaining the impact of practice testing.

Fiorella and Mayer (2015) explain the positive impact of practice testing as partially attributable to selecting relevant information from long-term memory: “Selecting involves activating relevant information from long-term memory that can be used to answer questions from the practice test,” (p. 102). Mayer (2021), however, explicitly defines selection as “attend[ing] to relevant material in the multimedia lesson” (p. 39). Mayer’s (2021) definition of selection contradicts Fiorella and Mayer’s (2015) explanation of practice testing. Either the process of selection must be expanded to include attending to relevant information in long-term memory as well as in multimedia lessons, or the two processes must be explicitly distinguished (i.e., “selection” to refer to attending to material in multimedia lessons, and “retrieval” to refer to attending to material in long-term memory).

The theoretical distinction between selection and retrieval has practical consequences. If the process of selecting information from multimedia lessons is conceptually distinct from the process of retrieving information from long-term memory, then a new set of principles to facilitate retrieval should be theoretically developed and empirically tested (practice testing is only one potential technique among many, including self-summarizing, self-explanation, and mnemonics). Attempting to apply existing principles designed to facilitate selection of information from multimedia lessons might prove ineffective, or even counterproductive, when used to facilitate retrieval of information from long-term memory. Meanwhile, effective techniques for facilitating retrieval of information from long-term memory might be overlooked because they fail to facilitate selection of information from multimedia lessons.

Retrieval is implied but not explained as a component of integration as well. Mayer (2021) defines integration as the synthesis of “created knowledge with other knowledge including knowledge brought in from long-term memory” (p. 41). This implies two processes: the synthesis of new and existing knowledge (i.e., integration), and the process of bringing-in existing knowledge from long-term memory (i.e., retrieval). This distinction between retrieval and integration is in line with the finding that self-explanation is most effective when prompted at the beginning of a lesson, which implies that these prompts work, at least in part, by facilitating retrieval of relevant existing knowledge prior to learning in order facilitate later integration with information subsequently presented during instruction (Bisra et al., 2018). Understanding the need to facilitate retrieval as well as integration, and effective techniques for doing so, could contribute valuable guidance to instructional designers.

### What is the relative impact of learning principles on selection and organization processes?

The organization of cognitive information plays a central role in many theories of learning (e.g., Ausubel, 1960; Bartlett, 1932). Some theories go so far as to claim that the key difference between novices and experts is that experts have cognitively organized information in such a way as to allow for fluid processing, whereas novice learners stumble through a series of arbitrary facts without understanding the relationships among them (Miller, 1956). Whereas other theories have relatively specific descriptions of how information is organized (e.g., Ausubel, 1960; Bartlett, 1932), CTML is vague on this point.

Interestingly, CTML attributes the positive effects of a number of principles primarily in terms of accommodations to cognitive capacity limitations, as opposed to facilitating the cognitive organization of information. For example, spatial and temporal contiguity are explained mainly in terms of reducing extraneous effort put towards visual search and reducing information decay in working memory (Mayer, 2021). While there is evidence to support those propositions, there is also evidence that visual and temporal proximity serve as cues to learners regarding the organization of information, facilitating the spontaneous creation of chunks (Oberaur et al., 2018). Chunking exponentially increases the amount of information a learner can process in working memory at a given time, meaning that the forming of chunks is essential to learning complex skills and mental processes (Cowan, 2001; Miller, 1956; Oberaur et al., 2018).

Organization likely plays a role in the positive impact of segmenting, as well. Within CTML, segmenting is often attributed to preventing decay of information in working memory (Mayer, 2021), but there is evidence that segmenting serves as a cue to underlying informational structure, and that learners tend to chunk information together that is presented within a segment (Spanjers et al., 2010). These are not mutually exclusive explanations, and both mechanisms likely play a role, but the relative impact of selection and organization have practical implications. As discussed above, one open question regarding segmenting is the ideal segment length. If segmenting serves mainly to reduce decay of information held in working memory, then ideal segment length should be determined by the rate of decay. If segmenting functions mainly by facilitating organization, however, then the length of a segment is not as important as the cohesiveness of the material within it. The majority of research on segmenting has focused on studying recall and transfer as learning outcomes (e.g., see Fiorella & Mayer, 2015; Mayer, 2021); future research should explore the impact of segmenting on organization as well.

### How do CTML principles impact learner satisfaction and engagement?

A final gap in the CTML research has to do not with the principles themselves, but the purported outcomes of the principles. While learning is prioritized as the most direct outcome of interest, authentic scenarios of online instruction/learning happen within broader organizational contexts (e.g., universities) where other learner outcomes such as satisfaction and engagement must be considered (e.g., Weerasinghe & Fernando, 2017). Even in the absence of a clear organizational context, learner satisfaction and engagement must be considered insofar as they also are likely to impact learning. Indeed, meta-analytic evidence has supported a significant positive relationship between learner reactions (i.e., learner satisfaction) and changes in declarative and procedural knowledge (Sitzmann et al., 2008).

Not only is consideration of learner satisfaction lacking in the CTML theoretical framework and research body, but certain CTML principles seem counter to such affective outcomes. For example, the coherence principle suggests that only directly relevant information be presented in an online multimedia lesson and specifically prohibits the inclusion of so-called ‘seductive details’ such as an attention-grabbing story or image that does not align with the target instructional content. Yet such attention-grabbing material is more likely to be perceived as enjoyable by learners and may contribute to their overall affective reactions to the lesson (Sung & Mayer, 2012). Such affective reactions have been highlighted as important factors to consider whenever designing multimedia learning materials (e.g., Um et al., 2012). Future research should explore such double-edged principles in order to account for a potentially contradictive set of learner outcomes (e.g., more learning but less satisfaction) to provide practical guidance to instructors needing to balance multiple objectives.

### Summary of CTML limitations and gaps

In sum, the application of learning principles derived from CTML to online multimedia lessons raises a number of important theoretical questions. We focused on four here: What is the nature of the relationship between working memory capacity and duration? Does retrieval need to be incorporated into CTML as a key learning process? What is the relative impact of learning principles on selection and organization processes? How do CTML principles impact learner satisfaction and engagement?

Conceptual clarification of these issues will not only provide better guidance to instructional designers as they use technology to incorporate learning principles into their lessons, it will improve the scientific understanding of learning as a whole. Further research is needed to address these questions and, in doing so, continue to strengthen the practical application of CTML.

## Conclusion

The purpose of this article was to support a reciprocal relationship between CTML and the practice of engaging in online learning/instruction. We approached this aim through a critical review of CTML, organized by a series of four inquiries. To address inquiry 1 (*What CTML principles are clearly supported by research and practically useful?*), the initial section summarized research on learning principles derived from CTML that are especially applicable to designing online multimedia lessons. In order to address inquiry 2 (*How can CTML principles be applied using commonly available online instruction/learning tools?*) we framed this summary in the context of practical guidance on how to apply these principles using commonly available software programs and websites. Specifically, we discussed how to use slide-sharing programs to create self-paced, narrated, animated segments, survey programs to interpolate questions and prompts between these segments to facilitate generative learning activities, and video-sharing sites to provide learners with control over relatively superficial aspects of instruction. We illustrated these principles with visual examples from our own authentic online learning scenarios (see Figs. 2, 3; Table 1). Throughout, the intent of this first section was to strengthen the science to practice connection relevant to CTML and effective online learning/instruction.

The latter section of this article focused on the practice to science connection relevant to CTML, and specifically how the application of this theory in a range of authentic online learning/instructional scenarios highlights important limitations with the framework and gaps in the surrounding research. Specifically, this second section targeted inquiry 3 (*What limitations exist when using CTML to guide the development of online multimedia lessons?*) and inquiry 4 (*What gaps exist in the current research surrounding CTML that would be most beneficial for practitioners?*). These limitations and gaps include the need to better understand the relationship between working memory capacity and duration, the importance of retrieval as a learning process, the relative impact of learning principles on selection and organization processes, and the impact of CTML principles on learner affective outcomes.

Online multimedia lessons have proven themselves to be a popular and effective learning tool, and CTML provides a number of theoretically sound principles that can be used to create them. By utilizing learning science to inform application *and* using practical application to strengthen science, we can continue to drive our theoretical understanding of learning while providing instructors practical guidance for creating effective online multimedia lessons.
